# Immunological Reprogramming in Cardiomyopathies: From Cardiomyocyte Injury to Disease of the Cardiac Immune Ecosystem

**DOI:** 10.3390/cells15141308

**Published:** 2026-07-22

**Authors:** Tomasz Urbanowicz, Krzysztof J. Filipiak

**Affiliations:** 1 Cardiac Surgery and Transplantology Department, Poznan University of Medical Sciences, ½ Długa, 61-848 Poznań, Poland; 2 The Centre of Postgraduate Medical Education, 99/103 Marymoncka Street, 01-813 Warsaw, Poland

**Keywords:** cardiomyopathies, heart failure, immunological reprogramming, cardiac immune ecosystem, innate immunity, immunometabolism, cGAS–STING, trained immunity, single-cell transcriptomics, immunophenotyping

## Abstract

**Highlights:**

1. This review proposes a conceptual shift from a cardiomyocyte-centered view of cardiomyopathies to a disease model grounded in dysregulation of the cardiac immune ecosystem.
2. Diverse immune mechanisms—including innate immune activation, immunometabolic remodeling, trained immunity, cGAS–STING signaling, and fibroblast–immune interactions—are integrated into a unified framework of chronic cardiac remodeling.
3. Emerging single-cell, spatial transcriptomic, and systems biology approaches support immunophenotyping as a complementary strategy to conventional phenotypic and genetic classification of cardiomyopathies.
4. Understanding cardiomyopathies as disorders of the cardiac immune ecosystem may facilitate mechanism-based patient stratification and support the future development of precision immunomodulatory therapies.

**Abstract:**

Cardiomyopathies have traditionally been regarded as disorders driven primarily by cardiomyocyte injury resulting from genetic defects, infection, metabolic stress, or toxic exposure. This paradigm has substantially advanced diagnosis and treatment. Still, it does not fully account for the marked heterogeneity in disease progression, persistent fibrosis, or variable therapeutic responses among patients with similar phenotypes. Increasing evidence indicates that immune remodeling is not merely a secondary consequence of myocardial injury but a dynamic process that actively shapes disease evolution. In this review, we integrate recent advances in cardiovascular immunology, single-cell and spatial transcriptomics, immunometabolism, and systems biology to propose a unified framework of immunological reprogramming in cardiomyopathies. We discuss how danger-associated molecular patterns, inflammasome activation, trained immunity, the cGAS–STING pathway, fibroblast–immune interactions, and the cardio–bone marrow axis converge to establish chronic inflammatory circuits that promote fibrosis, electrical remodeling, and progressive ventricular dysfunction. We further examine the emerging concept of immunotypes, emphasizing that distinct immune programs may underlie the biological heterogeneity of cardiomyopathies beyond conventional phenotypic or genetic classification. Finally, we discuss the translational potential of immune profiling, advancing a shift toward viewing cardiomyopathies as disorders of a dysregulated cardiac immune ecosystem. We propose that immune ecosystem organization constitutes an additional biological dimension that complements traditional phenotypic and genetic classifications of cardiomyopathies.

## 1. Introduction

Cardiomyopathies represent a heterogeneous group of myocardial disorders characterized by structural and functional abnormalities that frequently progress to heart failure, arrhythmias, and premature death [1,2]. Traditionally, their pathogenesis has been interpreted through a cardiomyocyte-centered paradigm, in which genetic mutations, viral injury, toxic exposure, or metabolic stress directly damage contractile cells and initiate myocardial remodeling [3,4]. This framework has been highly successful in identifying causal genes, defining disease phenotypes, and guiding contemporary therapies [5]. Nevertheless, it incompletely explains several fundamental clinical observations, including the remarkable heterogeneity of disease penetrance among carriers of identical pathogenic variants, the persistence of myocardial fibrosis despite optimal guideline-directed therapy, and the variable progression of heart failure in patients with apparently similar structural phenotypes [6,7].

Over the last decade, advances in single-cell transcriptomics, spatial transcriptomics, proteomics, and systems immunology have fundamentally changed our understanding of cardiac biology [8,9]. These technologies have revealed that the failing heart is not simply a collection of injured cardiomyocytes but a complex multicellular ecosystem composed of immune cells, fibroblasts, endothelial cells, stromal populations, and hematopoietic-derived cells that continuously interact through dynamic signaling networks [10,11]. Within this framework, disease progression is increasingly recognized as the consequence not only of the initiating injury but also of how the immune system interprets and responds to it.

A growing body of evidence indicates that innate immune activation is a central driver of cardiac remodeling across diverse cardiomyopathies [12,13]. Damage-associated molecular patterns (DAMPs) released from stressed or injured cardiomyocytes activate pattern-recognition pathways, including Toll-like receptors, the NLRP3 inflammasome, and the cGAS–STING axis [14,15,16]. These pathways promote recruitment and reprogramming of monocyte–macrophage populations, activation of fibroblasts, production of pro-inflammatory cytokines, and ultimately the development of fibrosis and electrical remodeling. Importantly, these mechanisms appear to converge across seemingly distinct etiologies, including dilated cardiomyopathy, arrhythmogenic cardiomyopathy, laminopathies, myocarditis, and metabolic heart disease [17,18,19].

Concurrently, emerging concepts such as immunometabolism, trained immunity, and clonal hematopoiesis of indeterminate potential (CHIP) have expanded the scope of cardiovascular immunology beyond the heart itself [20,21,22]. Immune cells can undergo long-lasting functional reprogramming via metabolic and epigenetic mechanisms, creating persistent pro-inflammatory states that continue to influence disease progression even after the original insult has resolved. Furthermore, bidirectional communication between the heart and the hematopoietic system has established the concept of a cardio–bone marrow axis, suggesting that chronic heart failure should be viewed as a systemic immunohematologic disorder rather than an isolated cardiac disease [23].

These discoveries challenge conventional classifications based solely on phenotype or genotype and support the emergence of a new conceptual framework centered on immunological remodeling. In this model, the disease trajectory is determined not only by the nature of myocardial injury but also by the dominant immune programs activated within the cardiac microenvironment. Such programs may define distinct “immunotypes” characterized by inflammasome activation, interferon signaling, macrophage-driven inflammation, fibroblast activation, or immunometabolic dysfunction. Recognition of these immunotypes may ultimately enable precision immunomodulatory therapies tailored to the biological mechanisms driving remodeling in individual patients.

In this review, we discuss the evolving concept of immunological reprogramming in cardiomyopathies and heart failure. We examine the cellular and molecular pathways linking cardiomyocyte injury to chronic immune activation, including DAMP signaling, inflammasome activation, trained immunity, immunometabolism, the cGAS–STING pathway, and cardio–bone marrow interactions. We further explore how recent advances in single-cell and spatial technologies are redefining our understanding of myocardial remodeling and discuss the potential transition from traditional phenotyping toward immunophenotyping as a foundation for future precision cardiovascular medicine.

Central hypothesis of this review:

We propose that the biological heterogeneity of cardiomyopathies is determined not only by the initiating myocardial injury but also by the manner in which the cardiac immune ecosystem interprets and sustains that injury. Although cardiomyocyte damage initiates disease, persistent immune reprogramming progressively becomes an independent determinant of remodeling, fibrosis, arrhythmogenesis, and heart failure progression. Consequently, individual differences in immune-cell composition, activation state, metabolic programming, and intercellular communication may explain why patients with apparently similar genetic or structural phenotypes often experience markedly different clinical trajectories. We therefore hypothesize that immunophenotyping may complement conventional phenotypic and genetic classification by identifying biologically distinct immune programs that support precision therapeutic strategies.

**Figure 1:**
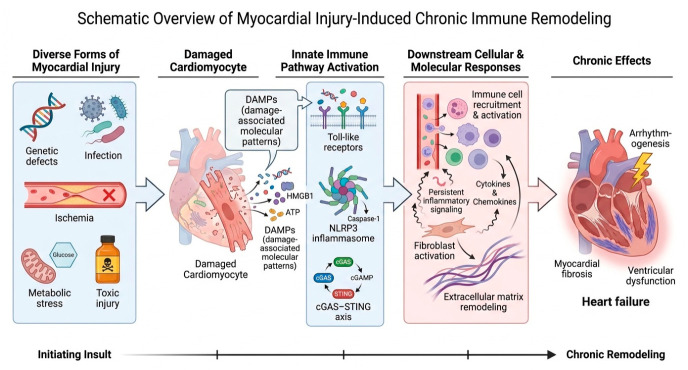
Immunological Reprogramming: From Cardiomyocyte Injury to Heart Failure. Abbreviations: ATP, adenosine triphosphate; cGAMP, cyclic guanosine monophosphate–adenosine monophosphate; cGAS, cyclic GMP–AMP synthase; DAMPs, damage-associated molecular patterns; HMGB1, high-mobility group box 1; NLRP3, NOD-like receptor family pyrin domain-containing 3; STING, stimulator of interferon genes; TLR, Toll-like receptor. Arrow notation: Solid black arrows indicate the sequential progression from initiating myocardial injury through innate immune activation, downstream cellular responses, and chronic cardiac remodeling. Arrows originating from DAMPs toward Toll-like receptors, the NLRP3 inflammasome, and the cGAS–STING pathway indicate activation of the principal innate immune sensing pathways. Arrows from these signaling pathways toward immune-cell recruitment, persistent inflammatory signaling, cytokine and chemokine production, fibroblast activation, and extracellular matrix remodeling denote propagation and amplification of immune-mediated tissue remodeling. The final arrows indicate progression toward myocardial fibrosis, ventricular dysfunction, arrhythmogenesis, and ultimately heart failure as the cumulative consequences of sustained immune dysregulation. Created with www.figureLabs.ai by Urbanowicz T. (2026), ID: FL-PUB-20260628-Q9LGNW.

The mechanisms discussed throughout this review are traditionally studied in separate experimental frameworks, yet disease progression arises from their combined activity rather than from any single pathway. To provide a unified conceptual perspective, Figure 1 summarizes the proposed sequence of events linking diverse forms of myocardial injury to chronic immune remodeling and its clinical consequences.

### Methodological Considerations

This review was developed through a comprehensive narrative analysis of the contemporary literature addressing the role of immune remodeling in cardiomyopathies and heart failure. Literature retrieval was performed primarily using the PubMed/MEDLINE database, with particular emphasis on studies published in the last decade. At the same time, seminal earlier investigations were included when essential to establishing mechanistic concepts or historical context.

The search strategy was designed to capture the evolving intersection between cardiovascular biology, immunology, and systems medicine. Search terms included, among others, cardiomyopathy, heart failure, cardiac inflammation, immune remodeling, immunological reprogramming, damage-associated molecular patterns (DAMPs), NLRP3 inflammasome, cGAS–STING, trained immunity, immunometabolism, cardiac macrophages, fibroblast–immune interactions, cardiac fibrosis, clonal hematopoiesis, cardio–bone marrow axis, single-cell transcriptomics, spatial transcriptomics, arrhythmogenesis, and immunophenotyping. These terms were queried individually and in multiple Boolean combinations to identify mechanistic, translational, and clinical studies relevant to immune-mediated cardiac remodeling.

Representative search strategies included combinations such as “cardiomyopathy AND immune remodeling”, “heart failure AND inflammation”, “NLRP3 inflammasome AND cardiac remodeling”, “cGAS–STING AND cardiomyopathy”, “trained immunity AND heart failure”, “immunometabolism AND cardiac inflammation”, “cardiac macrophages AND fibrosis”, “clonal hematopoiesis AND heart failure”, and “spatial transcriptomics AND cardiac remodeling”.

Priority was given to original experimental investigations, translational studies, landmark clinical observations, and high-impact reviews that contributed substantially to the current understanding of immune mechanisms in cardiovascular disease. Additional relevant publications were identified through manual examination of reference lists from key articles. Rather than providing an exhaustive, systematic synthesis, this review aimed to integrate emerging evidence from traditionally distinct fields—including innate immunity, immunometabolism, epigenetic reprogramming, hematopoietic biology, and spatial systems biology—into a unified conceptual framework for immunological remodeling in cardiomyopathies.

## 2. The Heart as an Immune Organ: Homeostasis and the Origins of Immune Remodeling

For many years, the immune system was considered relevant to cardiac pathology primarily in the context of infectious myocarditis, acute myocardial infarction, or systemic inflammatory disorders. This view has changed substantially with the recognition that the healthy heart contains a highly specialized immune network that contributes to tissue homeostasis under physiological conditions. Rather than functioning solely as responders to injury, resident immune cells actively participate in maintaining myocardial structure, metabolism, vascular integrity, and electrical function [24,25].

Among the most important components of this network are tissue-resident macrophages. In the healthy myocardium, resident CCR2-negative macrophages, many of which originate during embryonic development, perform a wide range of homeostatic functions [26,27]. These cells remove apoptotic debris through efferocytosis, support angiogenesis, contribute to extracellular matrix maintenance, and interact closely with cardiomyocytes and fibroblasts. Experimental studies have further demonstrated that resident macrophages participate in electrical conduction within the atrioventricular node, highlighting a previously unrecognized link between immunity and cardiac electrophysiology.

Cardiac immune homeostasis is additionally maintained by regulatory T lymphocytes (Tregs), which suppress excessive inflammatory activation and promote tissue repair [28,29]. Through the production of anti-inflammatory mediators, including interleukin-10 and transforming growth factor-β, Tregs contribute to immune tolerance within the myocardium and limit maladaptive remodeling following injury [30]. The importance of these populations is increasingly supported by experimental models demonstrating accelerated fibrosis and ventricular dysfunction following their depletion.

Equally important is the recognition that fibroblasts are not merely structural cells responsible for collagen production [31,32]. Contemporary single-cell studies have revealed that cardiac fibroblasts possess substantial immunological capabilities [33]. They express pattern-recognition receptors, including Toll-like receptors, that respond directly to cytokines and danger signals and actively shape leukocyte recruitment by secreting chemokines such as CCL2 and inflammatory mediators such as IL-6 [34,35]. Consequently, fibroblasts occupy a central position at the interface between tissue injury and immune activation.

Profound alterations in the cardiac immune landscape often accompany the transition from physiological homeostasis to pathological remodeling. One of the earliest detectable changes is the replacement of resident macrophage populations by inflammatory CCR2-positive monocyte-derived macrophages recruited from the circulation. This shift fundamentally alters the myocardium’s biological behavior. Whereas resident macrophages promote repair and tissue maintenance, infiltrating populations are characterized by increased production of pro-inflammatory cytokines, fibroblast activation, and amplification of extracellular matrix remodeling.

Importantly, these alterations frequently precede overt structural changes detectable by conventional imaging techniques. Experimental and translational studies suggest that immune reprogramming may occur long before the development of extensive fibrosis or measurable declines in ventricular function. Consequently, loss of immune homeostasis should be regarded not merely as a consequence of myocardial injury but as an early and potentially decisive event in the progression of cardiomyopathy.

These observations support a broader conceptual shift in cardiovascular biology. Rather than viewing the heart as an organ that becomes inflamed only after injury, it may be more appropriate to consider it a highly regulated immunological ecosystem in which disease emerges when physiological repair programs become chronically dysregulated. Understanding the mechanisms governing this transition is therefore essential for developing future therapeutic strategies to prevent maladaptive remodeling before irreversible structural damage occurs.

The cellular landscape of the myocardium extends far beyond cardiomyocytes, encompassing multiple immune and stromal populations that continuously interact under physiological and pathological conditions. Because these cells differ substantially in origin, function, and contribution to remodeling, a structured overview of their respective roles within the cardiac immune ecosystem is presented in Table 1.

**Figure 2:**
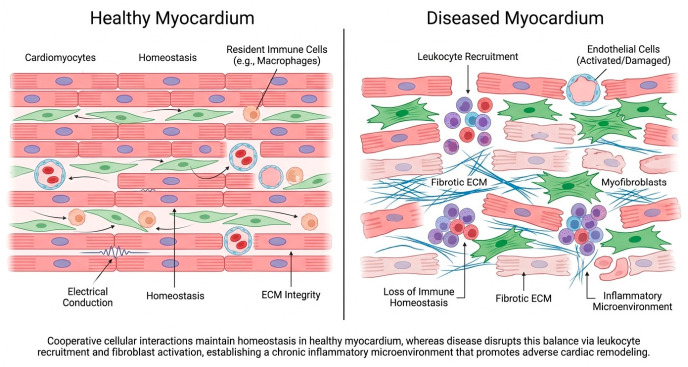
The Cardiac Immune Ecosystem in Homeostasis and Disease. Abbreviations: ECM—extracellular matrix. Activated microvessels facilitate leukocyte adhesion and transmigration into the myocardial interstitium during chronic immune remodeling. Black arrows indicate physiological intercellular communication and maintenance of tissue homeostasis. Microvessels illustrate the myocardial microcirculation, which coordinates leukocyte trafficking and intercellular communication within the cardiac immune ecosystem. Created by FigureLabs by Urbanowicz T. (2026), ID: FL-PUB-20260628-FOGCH0.

Understanding cardiomyopathy requires consideration not only of individual cell populations but also of the relationships among them. The transition from physiological immune surveillance to pathological remodeling involves coordinated changes across multiple cellular compartments. Figure 2 illustrates this shift within the myocardial ecosystem, emphasizing that alterations in cellular balance may precede overt structural disease.

Although this review primarily focuses on innate immune mechanisms, adaptive immunity also contributes to remodeling in selected cardiomyopathies. T-cell subsets, B-cell activation, and autoantibody-mediated responses have been implicated particularly in inflammatory cardiomyopathies and post-myocarditis remodeling. Interactions between adaptive and innate immune pathways likely influence disease chronicity, although their relative importance varies among cardiomyopathy phenotypes.

## 3. Damage-Associated Molecular Patterns: Translating Cardiomyocyte Injury into Innate Immune Activation

A central question in contemporary cardiovascular immunology is how the immune system detects myocardial injury in the absence of infection. Most cardiomyopathies develop without an identifiable pathogen, yet they exhibit many hallmarks of innate immune activation, including cytokine production, monocyte recruitment, inflammasome signaling, and progressive fibrosis [60]. The answer lies in the ability of injured cells to release endogenous danger signals collectively known as damage-associated molecular patterns (DAMPs) [61].

DAMPs represent intracellular molecules that are normally concealed from immune surveillance but become exposed or released following cellular stress, necrosis, mechanical injury, or mitochondrial dysfunction [62]. In the myocardium, these molecules include extracellular ATP, high-mobility group box 1 protein (HMGB1), mitochondrial DNA, nuclear DNA fragments, heat-shock proteins, extracellular RNA, and extracellular matrix degradation products. Although structurally diverse, these signals share a common function: they inform the immune system that tissue integrity has been compromised [63,64].

Among the various DAMPs identified in cardiac disease, mitochondrial components appear particularly important. Because mitochondria evolved from ancestral bacteria, mitochondrial DNA retains molecular characteristics resembling those of microbial nucleic acids [65]. When released into the extracellular space or cytoplasm, mitochondrial DNA is recognized by innate immune receptors as a potent danger signal. Similarly, extracellular ATP serves as an indicator of membrane disruption and cellular stress, whereas HMGB1 is released during necrosis and is a molecular marker of tissue damage.

An interconnected network of pattern-recognition receptors mediates recognition of DAMPs. Toll-like receptors, particularly TLR2, TLR4, and TLR9, play a prominent role in sensing extracellular and endosomal danger signals [66,67]. Cytoplasmic DNA activates the cGAS–STING pathway, while multiple forms of cellular stress converge on the NLRP3 inflammasome [68]. Together, these systems translate tissue injury into inflammatory signaling, leading to activation of nuclear factor-κB (NF-κB), production of cytokines, recruitment of monocytes, and stimulation of fibroblast activity.

An important consequence of this framework is that diverse etiologies may activate remarkably similar downstream pathways. Genetic cardiomyopathies caused by variants in TTN, LMNA, DSP, or FLNC, viral myocarditis, anthracycline-induced cardiotoxicity, ischemic injury, and metabolic stress all generate cellular damage capable of releasing DAMPs [60,69]. Consequently, the immune system responds not to the specific cause of injury but to its molecular consequences. This concept helps explain why distinct forms of cardiomyopathy frequently converge toward common pathological features, including chronic inflammation, fibrosis, and ventricular dysfunction.

Under physiological circumstances, DAMP-mediated signaling is transient and contributes to effective tissue repair. However, persistent or repetitive release of danger signals fundamentally alters this response. Continuous activation of innate immune pathways promotes sustained recruitment of inflammatory cells, prolonged fibroblast activation, and progressive extracellular matrix remodeling. Over time, the repair process becomes self-sustaining and partially independent of the initiating insult. This phenomenon may explain why many cardiomyopathies continue to progress even after viral clearance, stabilization of hemodynamic stress, or control of the original disease trigger.

## 4. NLRP3 Inflammasome: A Central Hub Linking Cellular Stress to Cardiac Remodeling

Among the innate immune pathways implicated in cardiovascular disease, the NLRP3 inflammasome has emerged as one of the most extensively studied molecular platforms connecting tissue injury with chronic inflammation and adverse remodeling. Although initially characterized as a key component of host defense against pathogens, increasing evidence suggests that NLRP3 also functions as a sensor of sterile tissue injury, making it particularly relevant to the pathogenesis of cardiomyopathies and heart failure.

The NLRP3 inflammasome is a multiprotein signaling complex composed of the sensor molecule NLRP3 (NOD-like receptor family pyrin domain-containing 3), the adaptor protein ASC (apoptosis-associated speck-like protein containing a CARD), and pro-caspase-1. Its activation is typically described as a two-step process [70,71]. In the diseased myocardium, multiple upstream triggers capable of activating NLRP3 coexist simultaneously. Mechanical stress, mitochondrial injury, ischemia, metabolic overload, and genetic defects that compromise cardiomyocyte integrity collectively create a cellular environment conducive to inflammasome activation. Importantly, NLRP3 signaling is not restricted to infiltrating immune cells. Cardiomyocytes, fibroblasts, endothelial cells, and resident macrophages have all been shown to participate in inflammasome-mediated signaling, underscoring the multicellular nature of myocardial inflammation [72,73]. One of the most significant consequences of inflammasome activation is the induction of pyroptosis, a highly inflammatory form of programmed cell death [74]. Unlike apoptotic cells, which generally do not elicit substantial immune activation, pyroptotic cells release intracellular contents, including cytokines and additional danger-associated molecular patterns, into the extracellular space. As a result, inflammasome activation not only responds to tissue injury but also amplifies it through self-reinforcing feed-forward mechanisms. This process may contribute to the transition from transient injury to persistent inflammation and progressive fibrosis observed in many forms of cardiomyopathy.

The translational relevance of these findings has been reinforced by clinical studies targeting downstream components of the inflammasome pathway. The CANTOS trial [75] provided the first large-scale evidence that selective inhibition of IL-1β can improve cardiovascular outcomes independently of lipid lowering, highlighting the pathogenic importance of inflammatory signaling in cardiovascular disease. Similarly, smaller studies using anakinra, an interleukin-1 receptor antagonist, have demonstrated favorable effects on inflammatory biomarkers and selected clinical endpoints in patients with heart failure and myocarditis [76,77]. Although these interventions do not directly inhibit NLRP3 itself, they provide indirect support for the concept that inflammasome-driven inflammation contributes to disease progression.

Despite considerable enthusiasm, several important questions remain unresolved. NLRP3 activation is a common downstream response to diverse forms of cardiac injury. Yet, the extent to which it functions as a primary driver versus a secondary amplifier of disease likely varies across cardiomyopathy subtypes. Moreover, chronic inhibition of innate immune pathways may carry unintended consequences, including impaired host defense and altered tissue repair. Consequently, future therapeutic strategies will likely require precise identification of patients in whom inflammasome signaling represents a dominant pathogenic mechanism.

Taken together, current evidence positions the NLRP3 inflammasome as a critical interface between cellular stress and maladaptive remodeling. Rather than acting as an isolated inflammatory pathway, NLRP3 integrates metabolic, mechanical, and immunological signals into a coordinated response that influences fibrosis, ventricular dysfunction, and arrhythmogenesis. Its central location within the network of cardiac immune remodeling makes it one of the most attractive yet also most challenging targets for future precision immunomodulatory therapies.

## 5. Immunometabolism: Metabolic Control of Cardiac Immune Responses

Rather than being passive consequences of activation, metabolic pathways actively determine immune cell phenotype and function. Over the past decade, immunometabolism has emerged as a fundamental regulator of innate and adaptive immune responses, providing a mechanistic link between cellular energetics and inflammation [78,79]. This concept is particularly relevant in cardiomyopathies and heart failure, where metabolic stress and immune activation frequently coexist.

Under physiological conditions, tissue-resident macrophages rely predominantly on oxidative phosphorylation and fatty-acid oxidation to support homeostatic functions, including efferocytosis, angiogenesis, and maintenance of tissue integrity. In contrast, inflammatory activation induces a profound metabolic shift toward aerobic glycolysis, a phenomenon analogous to the Warburg effect observed in cancer cells [80,81]. Although energetically less efficient, glycolysis enables rapid ATP generation and provides biosynthetic intermediates required for cytokine production and cellular proliferation.

Several interconnected signaling pathways, including mTOR, AMPK, HIF-1α, and NF-κB, orchestrate this metabolic reprogramming. Among these, HIF-1α has emerged as a particularly important regulator of inflammatory macrophage function [82]. Stabilization of HIF-1α promotes transcription of pro-inflammatory mediators, including IL-1β, while simultaneously reinforcing glycolytic metabolism. Consequently, metabolic and inflammatory signaling become tightly coupled processes that sustain one another through positive feedback mechanisms [83,84]. Elevated intracellular succinate stabilizes HIF-1α and enhances IL-1β production, effectively transforming a metabolic disturbance into an inflammatory signal. Such observations have fundamentally altered traditional views of metabolism, demonstrating that metabolites can function as signaling molecules with direct immunological consequences.

The clinical relevance of immunometabolism is supported by therapies originally developed for metabolic disease. Sodium–glucose cotransporter-2 inhibitors (SGLT2i) and glucagon-like peptide-1 receptor agonists (GLP-1RA) reduce cardiovascular events and heart-failure outcomes through mechanisms that extend beyond glycemic control, including effects on inflammation, oxidative stress, endothelial function, mitochondrial signaling, and immune-cell activation. These observations suggest that modulation of cellular metabolism may be an indirect yet clinically effective strategy to attenuate pathological immune activation [85,86,87]. These observations suggest that targeting cellular metabolism is an indirect but highly effective strategy for controlling pathological immune activation.

Collectively, accumulating evidence indicates that cellular metabolism is not simply a downstream consequence of immune activation but a fundamental determinant of immune-cell identity, functional polarization, and inflammatory behavior. Consequently, metabolic reprogramming is now recognized as a central mechanism regulating both innate and adaptive immune responses [88,89,90].

### Master Transcriptional Programs Orchestrating Immune Remodeling

Although individual inflammatory pathways are often discussed in isolation, they operate under the control of a relatively small set of transcriptional programs that integrate environmental stress into coordinated cellular responses [91]. Rather than functioning as isolated signaling cascades, innate immune activation, metabolic adaptation, oxidative stress, and tissue repair converge on master transcription factors that determine the magnitude, duration, and biological consequences of myocardial remodeling [92]. These regulatory networks translate diverse forms of cardiac injury into shared molecular programs, providing a plausible explanation for the remarkable convergence of genetically, metabolically, ischemically, and inflammation-distinct cardiomyopathies toward similar pathological phenotypes [93].

Among these regulators, hypoxia-inducible factor-1α (HIF-1α) occupies a central position. Although initially characterized as a sensor of oxygen deprivation, HIF-1α is now recognized as a broader integrator of cellular stress [94]. Persistent myocardial hypoxia resulting from microvascular dysfunction, increased wall stress, mitochondrial impairment, or extracellular matrix expansion promotes HIF-1α stabilization even in the absence of severe ischemia [95]. This phenomenon appears relevant across multiple cardiomyopathy phenotypes, including hypertrophic cardiomyopathy, diabetic cardiomyopathy, storage disorders, infiltrative diseases such as transthyretin amyloidosis, and cardiomyopathies associated with coronary microvascular dysfunction. Sustained HIF-1α activation promotes glycolytic reprogramming, mitochondrial dysfunction, angiogenic signaling, fibroblast activation, and inflammatory cytokine production, thereby linking metabolic adaptation with progressive structural remodeling [96].

An equally important regulator is nuclear factor-κB (NF-κB), which serves as a central coordinator of inflammatory gene expression [97]. Activation of Toll-like receptors, DAMP signaling, reactive oxygen species, inflammatory cytokines, and mechanical stress converges on NF-κB, resulting in transcription of numerous mediators involved in innate immunity, leukocyte recruitment, endothelial activation, and extracellular matrix remodeling [98]. Because NF-κB integrates signals originating from multiple upstream pathways—including NLRP3 inflammasome activation, cGAS–STING signaling, and oxidative stress—it represents one of the principal molecular hubs sustaining chronic myocardial inflammation [99]. Persistent activation of NF-κB further amplifies oxidative stress and inflammatory signaling, establishing self-reinforcing feed-forward circuits that promote fibrosis, electrical remodeling, and ventricular dysfunction [100].

Signal transducer and activator of transcription 3 (STAT3) illustrates the complexity of transcriptional regulation within the injured myocardium [101]. Unlike purely pro-inflammatory regulators, STAT3 exerts context-dependent effects that encompass cardiomyocyte survival, mitochondrial homeostasis, immune modulation, and tissue repair [102]. Transient STAT3 activation may support adaptive responses following acute injury, whereas chronic dysregulation has been associated with persistent inflammatory signaling, fibroblast activation, and maladaptive remodeling [103]. These observations emphasize that therapeutic intervention should not indiscriminately suppress inflammatory pathways but rather restore the physiological balance between protective and pathological transcriptional programs.

Additional regulatory systems further expand this network. Activator protein-1 (AP-1) contributes to inflammatory gene expression downstream of mitogen-activated protein kinase signaling. In contrast, SMAD transcription factors mediate transforming growth factor-β–dependent profibrotic responses that ultimately determine extracellular matrix accumulation and scar formation [104]. Rather than acting independently, HIF-1α, NF-κB, STAT3, AP-1, and SMAD signaling continuously interact through extensive transcriptional cross-talk, allowing metabolic stress, oxidative injury, immune activation, and mechanical loading to converge on a coordinated remodeling response [91]. Consequently, alterations in one regulatory pathway frequently influence multiple downstream biological processes simultaneously. Activation of these transcription factors ultimately regulates extensive networks of downstream effector genes controlling cytokine production, extracellular matrix turnover, mitochondrial adaptation, angiogenesis, and cellular survival.

Recognition of these master transcriptional programs has important therapeutic implications. Current guideline-directed therapies already modulate several of these pathways indirectly. Sodium-glucose cotransporter-2 inhibitors attenuate inflammatory activation by favorably affecting cellular metabolism, oxidative stress, and inflammasome activity. At the same time, glucagon-like peptide-1 receptor agonists influence immune-cell metabolism, endothelial function, and cytokine production [105]. Experimental strategies targeting HIF-1α, NF-κB, STAT3, or their downstream effector networks are also being actively investigated as potential approaches to interrupt maladaptive remodeling before irreversible fibrosis becomes established [106]. Although clinical translation remains challenging because these pathways regulate numerous physiological processes, targeting upstream transcriptional regulators offers the theoretical advantage of simultaneously modifying multiple interconnected mechanisms rather than inhibiting individual inflammatory mediators.

These observations suggest that transcriptional regulation represents a fundamental organizational level of cardiac immune remodeling. Master transcription factors do not merely control isolated signaling pathways but orchestrate the integrated biological response to myocardial injury. Understanding how these regulatory networks interact is therefore essential for the development of future precision immunomodulatory strategies that limit disease progression while preserving the physiological mechanisms of tissue repair.

## 6. Trained Immunity: Persistent Inflammatory Memory Beyond the Initial Injury

One of the most intriguing observations in cardiovascular medicine is the persistence of inflammatory activity long after the initiating insult has resolved. Patients recovering from viral myocarditis may continue to develop progressive ventricular dysfunction despite clearance of the infectious virus, reflecting the persistence of immune-mediated myocardial inflammation, adverse remodeling, and autoimmune mechanisms [107,108]. Similarly, Individuals carrying pathogenic variants associated with inherited cardiomyopathies frequently undergo prolonged periods of apparent clinical stability before experiencing age-dependent and sometimes abrupt disease progression characterized by ventricular dysfunction, arrhythmias, or heart failure [5,109]. These observations challenge the traditional view that immune activation is merely a transient response to tissue injury and suggest the existence of mechanisms capable of sustaining inflammation independently of the original trigger.

The concept of trained immunity has emerged as a potential explanation for this phenomenon [110,111]. Unlike adaptive immune memory, which relies on antigen-specific responses mediated by T and B lymphocytes, trained immunity describes a long-lasting functional reprogramming of innate immune cells and their progenitors. Following exposure to inflammatory stimuli, monocytes, macrophages, and hematopoietic stem cells undergo metabolic and epigenetic changes that enhance their responsiveness to subsequent challenges. As a result, future inflammatory responses become quantitatively and qualitatively different, even when triggered by unrelated stimuli [112,113]. Exposure to inflammatory mediators induces persistent modifications in chromatin accessibility, including histone acetylation and methylation events that facilitate transcription of pro-inflammatory genes. Among the best-characterized changes are increased H3K4 trimethylation and H3K27 acetylation at promoters and enhancers controlling cytokine production [114]. These alterations create a permissive transcriptional landscape that allows rapid reactivation of inflammatory programs upon subsequent stimulation.

Although direct evidence in human cardiomyopathies remains limited, several observations support the relevance of trained immunity in heart failure [115,116]. Patients with chronic heart failure frequently exhibit persistent elevations of inflammatory mediators despite the absence of active infection or overt tissue necrosis [117]. Moreover, circulating inflammatory biomarkers often remain elevated despite stabilization of hemodynamic status and optimization of guideline-directed medical therapy, suggesting that immune activation may become partially self-sustaining rather than simply reflecting ongoing hemodynamic stress [118,119]. Experimental studies further demonstrate that prior inflammatory stimulation induces innate immune memory, resulting in exaggerated inflammatory responses and accelerated adverse cardiac remodeling following subsequent cardiovascular insults [120,121]. The concepts of trained immunity provide a conceptual framework linking transient myocardial injury to sustained immune activation. It offers a mechanistic explanation for why inflammation frequently persists after the resolution of the initiating insult and suggests that the progression of cardiomyopathy may be determined not only by the extent of cardiac damage but also by the immune system’s capacity to remember and perpetuate that damage.

### Epigenetic Remodeling: From Adaptive Plasticity to Irreversible Cardiac Remodeling

One of the most fundamental yet incompletely understood questions in cardiomyopathy concerns the transition from reversible myocardial injury to irreversible structural disease. Clinical observations consistently demonstrate that some patients recover ventricular function following removal of the initiating insult. In contrast, others continue to progress toward diffuse fibrosis, electrical instability, and end-stage heart failure despite elimination of the original trigger and optimal medical therapy [122]. These divergent trajectories suggest that disease progression cannot be explained solely by the magnitude of myocardial injury but instead reflects the establishment of stable biological programs that progressively lose their capacity for physiological adaptation [123].

Epigenetic remodeling has emerged as a plausible mechanism underlying this transition. Unlike genetic mutations, epigenetic modifications alter gene expression without changing the underlying DNA sequence, allowing cells to acquire persistent functional phenotypes in response to environmental stimuli. DNA methylation, histone modifications, chromatin remodeling, and non-coding RNAs collectively determine transcriptional accessibility, thereby shaping cellular identity over prolonged periods [124]. Initially, these mechanisms facilitate adaptive responses to mechanical stress, oxidative injury, inflammation, and metabolic disturbance [125]. However, persistent activation of these regulatory programs may ultimately stabilize pathological cellular states that continue to drive remodeling independently of the initiating stimulus [126].

Although the concept of trained immunity illustrates this phenomenon in innate immune cells, similar principles appear to govern multiple cellular populations within the failing myocardium. Cardiomyocytes exposed to sustained inflammatory and metabolic stress undergo widespread transcriptional reprogramming characterized by altered calcium-handling proteins, mitochondrial dysfunction, impaired energy metabolism, and activation of fetal gene programs [127]. Increasing evidence further suggests that mitochondrial epigenetic regulation contributes to persistent metabolic dysfunction, limiting the ability of injured cardiomyocytes to restore normal bioenergetic homeostasis even after hemodynamic conditions improve [128].

Fibroblasts provide the most compelling example of persistent epigenetic reprogramming during cardiac remodeling [129]. Under physiological conditions, fibroblast activation is tightly regulated and resolves following completion of tissue repair. Chronic inflammatory signaling, however, promotes stable epigenetic changes that maintain myofibroblasts in a matrix-producing, contractile, and highly secretory phenotype [130]. Once established, these cells continue to synthesize extracellular matrix proteins, profibrotic cytokines, and matricellular mediators despite progressive separation from the initiating injury. Such epigenetic stabilization may explain why myocardial fibrosis frequently progresses even after effective treatment of the underlying disease.

Endothelial cells likewise develop persistent phenotypic alterations under chronic inflammatory conditions [131]. Sustained exposure to oxidative stress and inflammatory cytokines promotes endothelial dysfunction characterized by impaired nitric oxide bioavailability, increased leukocyte adhesion, and persistent activation of inflammatory signaling pathways [132]. This phenomenon, often referred to as endothelial memory, may contribute to persistent microvascular dysfunction and recruitment of inflammatory cells despite partial restoration of systemic cardiovascular homeostasis.

Importantly, these epigenetic programs do not develop independently within individual cell populations. Rather, reciprocal communication among cardiomyocytes, fibroblasts, endothelial cells, and immune cells continuously reinforces pathological transcriptional states through cytokines, growth factors, extracellular vesicles, metabolic intermediates, and matrix-derived signaling molecules [133]. As a consequence, the myocardium progressively transitions from a biologically plastic tissue capable of adaptive repair to a self-sustaining pathological ecosystem in which multiple cell populations become epigenetically synchronized in maintaining chronic inflammation and fibrosis [134,135].

Recognition of these mechanisms has important therapeutic implications. Conventional heart failure therapies primarily attenuate neurohormonal activation and hemodynamic stress but may only partially influence the epigenetic programs that perpetuate disease progression. Future therapeutic strategies may therefore require interventions that restore cellular plasticity rather than suppress individual inflammatory mediators [136]. Advances in epigenetic pharmacology, chromatin-targeted therapies, RNA-based therapeutics, and precision immunomodulation raise the possibility that pathological transcriptional programs may eventually become reversible before extensive structural remodeling has become permanently established [137].

The epigenetic remodeling may represent a critical biological interface between transient myocardial injury and irreversible cardiomyopathy. Rather than serving as passive consequences of chronic inflammation, epigenetic mechanisms progressively stabilize pathological cellular phenotypes throughout the cardiac immune ecosystem, ultimately determining whether myocardial injury resolves through adaptive repair or progresses to permanent fibrosis, electrical instability, and end-stage heart failure.

## 7. The cGAS–STING Axis: Interferon Signaling as a Driver of Chronic Cardiac Remodeling

While inflammasome activation primarily links tissue injury to interleukin-mediated inflammation, an additional innate immune pathway has emerged as a critical regulator of chronic sterile inflammation in the heart. The cyclic GMP–AMP synthase (cGAS)–stimulator of interferon genes (STING) pathway functions as a cytosolic DNA-sensing system that evolved to detect viral and bacterial nucleic acids [138,139]. However, increasing evidence indicates that this pathway also plays a central role in non-infectious cardiovascular disease by recognizing endogenous DNA released during cellular stress and injury.

Under physiological conditions, DNA is compartmentalized within the nucleus and mitochondria. The appearance of DNA within the cytoplasm is therefore interpreted by the cell as a danger signal. Cytoplasmic DNA is detected by cGAS, which catalyzes the production of cyclic GMP–AMP (cGAMP), a second messenger that activates STING localized within the endoplasmic reticulum membrane [140]. Activated STING recruits and activates TBK1, leading to phosphorylation of IRF3 and subsequent transcription of type I interferons and interferon-stimulated genes. In parallel, STING activates the NF-κB signaling pathway, thereby amplifying the expression of pro-inflammatory cytokines, chemokines, and other inflammatory mediators [141].

Among inherited cardiomyopathies, laminopathies provide the most compelling example of this pathway’s pathological relevance. The LMNA gene encodes nuclear lamins that maintain the structural integrity of the nuclear envelope. Pathogenic variants disrupt nuclear architecture, resulting in increased susceptibility to mechanical stress and the formation of transient nuclear envelope ruptures. Experimental studies have demonstrated that LMNA deficiency promotes nuclear envelope rupture and cytoplasmic DNA accumulation, which can activate cGAS–STING signaling. In several experimental models, this response has been associated with type I interferon signaling, inflammatory cell recruitment, myocardial fibrosis, and ventricular dysfunction. However, the contribution of cGAS–STING signaling to disease progression appears to be model-dependent and remains an area of active investigation [142,143].

The cGAS–STING pathway serves as a critical molecular bridge between cellular damage and chronic immune activation. Its ability to translate structural abnormalities, mitochondrial dysfunction, and tissue stress into sustained interferon signaling positions it as a central component of the emerging concept of immunological remodeling. Furthermore, the pathway provides a compelling example of how distinct cardiomyopathy phenotypes may be driven by specific immune programs, supporting the broader notion that future classification systems may need to incorporate immunological mechanisms alongside conventional phenotypic and genetic descriptors.

Future myocardial tissue characterization will likely integrate conventional histopathology, immunohistochemistry, ultrastructural electron microscopy, laser-capture microdissection, single-cell and spatial transcriptomics, proteomics, and multi-omic profiling to generate biologically informed disease classifications and guide individualized immunomodulatory therapies. Rather than replacing existing diagnostic approaches, these complementary technologies are expected to provide progressively higher-resolution characterization of myocardial biology, enabling identification of disease-defining cellular interactions, dominant immune programs, and patient-specific therapeutic targets. Such integrated tissue profiling may ultimately transform endomyocardial biopsy from a predominantly diagnostic procedure into a comprehensive platform for biological classification, prognostic stratification, and precision therapeutic decision-making.

## 8. The Cardio–Bone Marrow Axis and Clonal Hematopoiesis: Expanding the Boundaries of Cardiac Remodeling

For decades, research in heart failure and cardiomyopathy focused almost exclusively on the myocardium. Cardiomyocytes, fibroblasts, extracellular matrix remodeling, and neurohormonal activation were viewed as the principal determinants of disease progression.

Recent studies suggest that chronic cardiac remodeling cannot be understood solely as a local myocardial process. Instead, the failing heart appears to engage in continuous communication with the hematopoietic system, particularly the bone marrow, creating a bidirectional network that sustains inflammation long after the initiating injury [23]. In this model, the heart is not merely the recipient of immune responses but an active participant in shaping them.

Experimental studies have demonstrated that injured myocardium releases a diverse repertoire of cytokines, chemokines, damage-associated molecular patterns (DAMPs), growth factors, and neurohumoral mediators that influence hematopoietic stem and progenitor cell activity, promoting emergency myelopoiesis and sustained inflammatory responses within the bone marrow [144,145]. Cytokines, chemokines, damage-associated molecular patterns (DAMPs), and neurohumoral mediators released from the injured myocardium reach the bone marrow, where they alter the behavior of hematopoietic stem and progenitor cells. This signaling promotes emergency myelopoiesis, expands inflammatory myeloid progenitors, and increases the production and mobilization of pro-inflammatory monocytes that subsequently infiltrate the myocardium, thereby reinforcing chronic immune activation and adverse cardiac remodeling [146,147]. These cells subsequently traffic back to the myocardium, where they contribute to cytokine production, fibroblast activation, extracellular matrix remodeling, and progressive ventricular dysfunction. Once established, this circuit can maintain pathological remodeling even after the original trigger has diminished or disappeared.

Clinical observations support this interpretation. Imaging studies have identified increased metabolic activity within the bone marrow of individuals with advanced cardiovascular disease [148,149]. It may represent a previously underappreciated component of the disease process itself.

The importance of hematopoietic biology became even more apparent with the recognition of clonal hematopoiesis of indeterminate potential (CHIP) [150,151]. Initially regarded as an age-related hematologic phenomenon with uncertain clinical significance, CHIP has emerged as one of the most compelling links between immune biology and cardiovascular disease. Large population studies consistently demonstrated that individuals carrying CHIP-associated mutations experience substantially higher rates of cardiovascular events, heart failure, and cardiovascular mortality, independent of traditional risk factors [152,153].

The implications extend far beyond cardiovascular risk prediction. CHIP provides direct evidence that the biological characteristics of the hematopoietic system can influence the progression of cardiac disease. Two patients with comparable myocardial injury may experience markedly different outcomes because their immune systems respond differently to the same stimulus. From this perspective, part of the heterogeneity traditionally attributed to chance, environmental exposure, or incomplete penetrance may instead reflect differences in immune programming.

This concept aligns naturally with emerging ideas surrounding trained immunity. Both frameworks suggest that cardiovascular disease progression is shaped not only by the magnitude of injury but also by the state of the immune system responding to that injury. The myocardium and the hematopoietic compartment, therefore, become components of a single adaptive network rather than independent biological entities.

Although the pathways discussed in the preceding sections are often presented individually, they operate as components of an interconnected network linking cellular injury to chronic remodeling. Table 2 provides an integrated overview of the principal immune mechanisms implicated in cardiomyopathies, highlighting their key triggers, biological consequences, and potential relevance to disease progression.

**Figure 3:**
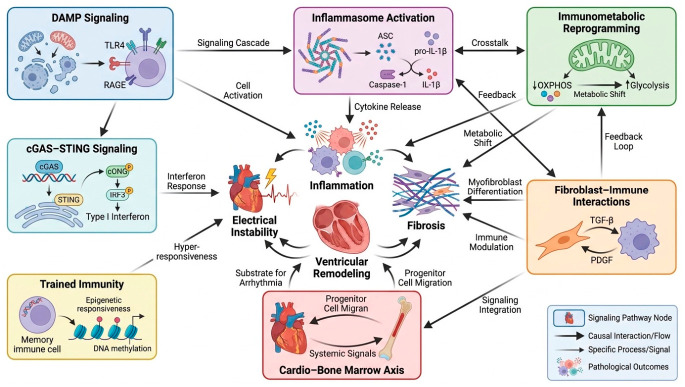
Integrated Network of Immune Remodeling Pathways. Abbreviations: ASC, apoptosis-associated speck-like protein containing a CARD; cGAMP, cyclic guanosine monophosphate–adenosine monophosphate; cGAS, cyclic GMP–AMP synthase; CHIP, clonal hematopoiesis of indeterminate potential; DAMPs, damage-associated molecular patterns; HIF-1α, hypoxia-inducible factor-1α; HMGB1, high-mobility group box 1; IL, interleukin; IRF3, interferon regulatory factor 3; mTOR, mechanistic target of rapamycin; NF-κB, nuclear factor kappa B; NLRP3, NOD-like receptor family pyrin domain-containing 3; ROS, reactive oxygen species; STING, stimulator of interferon genes; TBK1, TANK-binding kinase 1; TGF-β, transforming growth factor-β; TLR, Toll-like receptor; TNF-α, tumor necrosis factor-α. Arrow notation: Solid black arrows indicate activation or propagation of signaling between interconnected immune pathways involved in myocardial remodeling. Converging arrows illustrate the integration of DAMP signaling, NLRP3 inflammasome activation, immunometabolic remodeling, trained immunity, cGAS–STING signaling, the cardio–bone marrow axis, clonal hematopoiesis (CHIP), and fibroblast–immune cell crosstalk into a coordinated network of chronic inflammation. Bidirectional arrows indicate reciprocal communication and positive feedback among immune cells, stromal cells, and hematopoietic compartments, thereby amplifying inflammatory signaling. Downstream arrows denote progression toward persistent immune activation, extracellular matrix remodeling, myocardial fibrosis, ventricular dysfunction, electrical remodeling, and heart failure. Circular or feedback arrows (where present) represent self-reinforcing inflammatory circuits that sustain pathological remodeling independently of the initiating myocardial injury. Created with www.figurelabs.ai by Urbanowicz T. (2026), ID: FL-PUB-20260628-V9153L.

Individual mechanisms are often discussed separately for clarity, but substantial biological overlap exists between inflammatory, metabolic, and hematopoietic pathways involved in cardiac remodeling. Figure 3 highlights the interconnected nature of these processes and illustrates how apparently distinct mechanisms converge on common downstream remodeling programs.

Whether this paradigm will ultimately reshape clinical practice remains uncertain. Nevertheless, it compels a broader view of cardiomyopathy and heart failure. The biological drivers of disease may reside not only within the myocardium but also within distant tissues that continuously influence the composition and function of the immune response. Recognizing this possibility may prove essential for understanding why patients with seemingly similar cardiac phenotypes often follow remarkably different clinical courses.

## 9. Fibroblasts as Integrators of Immune Remodeling in Cardiomyopathy

Myocardial fibrosis is one of the most powerful determinants of clinical outcome across the spectrum of cardiomyopathies [164,165]. Regardless of etiology, the extent of fibrotic remodeling is closely associated with ventricular dysfunction, arrhythmia susceptibility, progression to heart failure, and mortality [166,167]. Yet despite its clinical importance, fibrosis has often been viewed primarily as a structural consequence of injury rather than as an active biological process. This perspective is increasingly difficult to reconcile with contemporary insights into cardiac remodeling.

Historically, fibroblasts were regarded as relatively passive cells responsible for maintaining extracellular matrix integrity and producing collagen in response to tissue damage. Recent advances in single-cell transcriptomics, spatial biology, and lineage-tracing studies have fundamentally altered this view. Fibroblasts are now recognized as highly dynamic cellular populations capable of sensing environmental signals, interacting with immune cells, and orchestrating tissue responses to injury [168,169]. In many respects, they function as integrators of information derived from mechanical stress, metabolic disturbance, and immune activation.

The relationship between fibroblasts and the immune system is a central determinant of chronic cardiac remodeling. Cardiac fibroblasts express a broad repertoire of pattern-recognition receptors, including Toll-like receptors, as well as cytokine and chemokine receptors that enable them to detect inflammatory and danger-associated signals within the injured myocardium [170]. Exposure to IL-1β, TNF-α, interferons, and transforming growth factor-β induces profound phenotypic reprogramming, promoting extracellular matrix synthesis and the secretion of chemokines, cytokines, and other inflammatory mediators that amplify local immune responses [171]. Consequently, fibroblasts should no longer be regarded as passive structural cells; rather, they function as active immunoregulatory cells that both respond to and perpetuate chronic myocardial inflammation through reciprocal communication with resident and infiltrating immune populations.

From a pathophysiological perspective, fibroblasts occupy a unique position within the remodeling cascade. Unlike immune cells, they persist within the tissue for prolonged periods and directly determine the structural consequences of inflammation. They therefore serve as the final common pathway through which diverse upstream signals—including inflammasome activation, interferon signaling, metabolic stress, and trained immunity—translate into permanent alterations in myocardial architecture. In this sense, fibroblasts may be viewed as the principal executors of immunological remodeling. Understanding how fibroblast populations interact with immune and stromal cells may therefore prove essential for developing future therapies that interrupt the transition from adaptive repair to irreversible myocardial fibrosis.

Persistent immune remodeling influences not only the severity but also the spatial distribution of myocardial injury [172]. Rather than remaining confined to the site of the initiating insult, chronic inflammatory signaling progressively reshapes the surrounding tissue through reciprocal interactions among activated fibroblasts, immune cells, endothelial cells, and cardiomyocytes. Local production of cytokines, chemokines, reactive oxygen species, and profibrotic mediators establishes inflammatory niches that extend beyond the original area of damage, promoting gradual expansion of extracellular matrix remodeling and regional alterations in tissue architecture [173]. These changes are accompanied by disruption of gap junction integrity, heterogeneous slowing of conduction, impaired electrical coupling, and increasing anisotropy, thereby creating a substrate favorable for both atrial and ventricular arrhythmias [174,175]. Consequently, inflammatory remodeling should not be regarded solely as a mechanism of fibrosis but also as a dynamic process governing the progressive propagation of electrical instability throughout the myocardium [176]. This concept provides a mechanistic explanation for the development of atrial fibrillation and ventricular tachyarrhythmias, as well as the increasing complexity of arrhythmogenic substrates observed during disease progression, even in the absence of new primary myocardial injury. Thus, persistent innate immune activation not only intensifies local injury but also drives the regional expansion of pathological substrates that promote atrial and ventricular arrhythmogenesis.

Ultimately, prospective clinical studies will be required to determine whether immune profiling provides incremental prognostic information beyond existing clinical, imaging, and genetic risk models.

## 10. Beyond Phenotype and Genotype: Toward an Immunological Classification of Cardiomyopathy

The remarkable heterogeneity of cardiomyopathies remains one of the most difficult problems in contemporary cardiovascular medicine. Despite extraordinary advances in molecular genetics, imaging, and heart failure therapeutics, our ability to predict disease behavior in individual patients remains limited. This is particularly evident in inherited cardiomyopathies, where carriers of the same pathogenic variant may exhibit profoundly different clinical trajectories. Some remain asymptomatic throughout life, whereas others develop early heart failure, malignant ventricular arrhythmias, or require transplantation. Similar observations can be made in acquired disease. Patients with apparently comparable degrees of ventricular dysfunction often demonstrate markedly different rates of progression despite receiving identical medical therapy.

Traditionally, inflammation has been viewed as a secondary consequence of myocardial injury. However, findings from single-cell transcriptomics, spatial biology, and experimental immunology increasingly challenge this interpretation [177,178]. Rather than representing a uniform reaction to tissue damage, immune activation appears highly heterogeneous, both between and within cardiomyopathy subtypes. Distinct inflammatory programs can be identified despite similar structural phenotypes, suggesting that the biological consequences of injury depend not only on the initiating insult but also on how the immune system interprets it.

This concept is most apparent in dilated cardiomyopathy. Although DCM is commonly classified as a single disease entity, it likely represents a final common phenotype arising from multiple biological processes [33,179]. Such findings suggest that DCM should not be viewed as a single disease but rather as a spectrum of pathological repair responses converging on a common anatomical phenotype.

Increasing experimental and translational evidence indicates that innate immune activation is an integral component of disease progression in arrhythmogenic cardiomyopathy [33,180]. Recurrent inflammatory episodes, activation of innate immune signaling pathways, and infiltration of inflammatory cells have been documented in both experimental models and human myocardial tissue, suggesting that immune dysregulation contributes to ongoing myocardial injury and fibrofatty remodeling [181,182]. Whether inflammation represents a primary pathogenic mechanism or a secondary amplifier of desmosomal dysfunction remains an area of active investigation. Nevertheless, the close temporal relationship between inflammatory activity and disease progression supports the concept that immunological mechanisms contribute substantially to the clinical phenotype and may represent future therapeutic targets.

At the opposite end of the spectrum lies HFpEF [183]. Unlike inherited cardiomyopathies, HFpEF rarely originates from a single dominant myocardial insult. Instead, it emerges from the cumulative effects of obesity, hypertension, diabetes, chronic kidney disease, aging, and systemic metabolic dysfunction [184]. What unites these diverse conditions is chronic low-grade inflammation [185]. In many respects, HFpEF may represent the clearest example of a disease in which immune activation is not a consequence of cardiac pathology but one of its principal drivers [186,187]. The myocardium becomes the target organ of a broader systemic process involving immune cells, adipose tissue, the microvasculature, and altered cellular metabolism.

These observations suggest that the future classification of cardiomyopathies may require expansion beyond conventional phenotypic and genetic frameworks. This does not imply that existing classifications are obsolete. On the contrary, genotype and phenotype remain essential determinants of diagnosis and management. However, they provide only a partial description of disease biology. Similar structural phenotypes may emerge from fundamentally different biological processes. Comparing cardiomyopathies through an immunological lens reveals important differences in dominant inflammatory programs, cellular responses, and remodeling pathways that are not readily apparent from conventional phenotypic classifications alone. Table 3 illustrates these emerging disease-specific patterns.

The proposed immunotypes should be viewed as heuristic frameworks rather than discrete disease categories. In practice, inflammatory, interferon-driven, immunometabolic, and profibrotic programs frequently coexist within the same myocardium, and their relative contribution may change during disease progression. Consequently, immunotypes are unlikely to represent fixed biological states. Instead, they may reflect dominant immune programs operating within a dynamic remodeling continuum. Future integration of single-cell, spatial, and longitudinal clinical datasets will be required to determine whether these proposed categories possess sufficient biological stability and prognostic value to support clinical implementation.

Importantly, the proposed framework does not imply that all cardiomyopathies originate from primary immune dysregulation. Rather, immune activation may represent either the initiating pathogenic mechanism, as in inflammatory cardiomyopathies, or a secondary process that progressively becomes an autonomous driver of disease progression after genetically, metabolically, mechanically, or toxic-induced myocardial injury.

Different immunotypes will probably require distinct combinations of circulating biomarkers, imaging characteristics, endomyocardial biopsy findings, spatial transcriptomics, and multi-omic profiling. Integration of these complementary datasets, rather than reliance on any single biomarker, will likely be necessary to accurately identify the dominant biological processes operating in individual patients and to support personalized immunomodulatory therapy.

An additional dimension may be required—one that captures the dominant immune processes operating within the myocardium. Whether such processes ultimately prove robust enough to justify formal immunological classification remains uncertain. Nevertheless, emerging technologies are increasingly capable of identifying distinct cellular and molecular signatures within diseased hearts. It is therefore conceivable that future patient stratification will incorporate not only genotype and ventricular phenotype but also immune state, fibrotic activity, and cellular network architecture.

The contribution of innate immunity differs substantially among cardiomyopathy subtypes. In inflammatory cardiomyopathies, including viral myocarditis and immune-mediated myocardial diseases, innate immune activation frequently initiates the pathogenic cascade leading to subsequent tissue injury [205,206]. In contrast, inherited, metabolic, toxic, pressure-overload, and infiltrative cardiomyopathies primarily arise from non-immunological insults, whereas innate immune activation develops secondarily in response to cardiomyocyte stress and progressively evolves into a self-sustaining amplifier of myocardial remodeling [207]. Consequently, although the temporal sequence differs across disease entities, persistent innate immune activation ultimately converges on common biological pathways that promote fibrosis, electrical remodeling, and ventricular dysfunction [208].

The most important implication of this shift is conceptual rather than terminological. Cardiomyopathies may differ not only because the myocardium is injured in different ways, but also because the immune system responds to that injury differently. Understanding those differences may ultimately prove as important as identifying the initiating cause itself.

We therefore propose that variability in immune-cell composition, immune activation, stromal remodeling, and intercellular communication contributes substantially to the clinical heterogeneity observed among patients with apparently similar cardiomyopathy phenotypes.

Different immunotypes will probably require distinct combinations of circulating biomarkers, advanced imaging, endomyocardial biopsy findings, spatial transcriptomics, proteomics, and other multi-omic approaches. Integration of these complementary datasets, rather than reliance on any single biomarker, will likely be necessary to accurately identify the dominant biological processes operating within individual patients and support personalized immunomodulatory therapy. Ultimately, prospective clinical studies will be required to determine whether immune profiling provides incremental prognostic information beyond existing clinical, imaging, and genetic risk models.

## 11. Spatial Biology of Cardiac Remodeling: From Cellular Composition to Cellular Geography

For many years, our understanding of myocardial remodeling was constrained by a fundamental limitation. We could identify the cells present within diseased myocardium, quantify fibrosis, characterize inflammatory infiltrates, and measure gene expression. What remained largely inaccessible was the spatial organization of these processes. Yet remodeling is inherently a spatial phenomenon. Fibrosis develops in specific regions rather than uniformly throughout the ventricle. Inflammatory infiltrates accumulate around particular structures. Arrhythmogenic substrates emerge in discrete anatomical locations. The critical question was never which cells are present within the failing heart, but where they are located and with whom they interact. Recent advances in spatial transcriptomics have begun to address this question and, in doing so, have substantially altered our understanding of cardiac pathology. Classical histology and bulk transcriptomic analyses provided valuable information regarding tissue composition but inevitably averaged biological signals across heterogeneous cellular populations [209,210]. Even single-cell sequencing, despite its transformative impact on cardiovascular biology, largely dissociates cells from their native anatomical context. Spatial approaches bridge this gap by integrating gene expression profiles with tissue architecture, enabling direct visualization of biological processes within intact myocardial microenvironments [211].

One of the most important insights emerging from these studies is that remodeling does not occur diffusely throughout the myocardium. Instead, it appears to be concentrated within highly organized cellular neighborhoods characterized by recurrent interactions among immune cells, fibroblasts, endothelial cells, and cardiomyocytes. These microenvironments are not random accumulations of cells but rather structured biological units in which local signaling networks shape disease progression.

Importantly, spatial biology provides an experimental framework for the concept of immunotypes. Much of the discussion surrounding immune heterogeneity in cardiomyopathies has remained inferential, based on circulating biomarkers, animal models, or dissociated-cell analyses. Spatial transcriptomics offers the opportunity to visualize these biological programs directly within the diseased myocardium. Rather than defining immunotypes solely through systemic measurements, future studies may identify them as distinct spatially organized ecosystems characterized by specific cellular compositions, signaling pathways, and patterns of tissue interaction.

Viewed in this context, the future challenge is no longer limited to identifying the molecules responsible for remodeling. Equally important will be understanding where these signals originate, how they propagate through tissue networks, and why certain cellular neighborhoods evolve toward fibrosis, arrhythmogenesis, or ventricular dysfunction while others remain relatively protected. Spatial biology has begun to reveal this previously hidden dimension of myocardial disease and, in doing so, has provided one of the strongest arguments for rethinking cardiomyopathies as disorders of a dysregulated cellular ecosystem rather than isolated abnormalities of cardiomyocytes alone.

## 12. Inflammation and Arrhythmogenesis: The Immunological Substrate of Electrical Remodeling

For much of the modern era of electrophysiology, ventricular arrhythmias were viewed primarily as structural diseases. Fibrosis, scar formation, chamber dilatation, and abnormalities of impulse conduction constituted the principal framework through which arrhythmogenesis was interpreted. This paradigm remains fundamentally valid and continues to explain a substantial proportion of clinical observations. Nevertheless, it leaves several important phenomena insufficiently explained.

Patients with active myocarditis may develop sustained ventricular arrhythmias before the appearance of extensive fibrosis [212,213]. Individuals with arrhythmogenic cardiomyopathy frequently experience periods of heightened arrhythmic activity that appear disproportionate to structural progression [214]. Similar clinicopathological dissociation has been observed in laminopathies and inflammatory cardiomyopathies, where electrical instability and malignant ventricular arrhythmias may precede substantial deterioration in ventricular function or the development of advanced structural remodeling [215]. Temporal changes in inflammatory biomarkers frequently coincide with periods of increased arrhythmic activity, suggesting that active myocardial inflammation may better reflect the dynamic arrhythmogenic substrate than static measures of structural remodeling [216,217]. These observations suggest that inflammation is not simply creating the substrate for future arrhythmias. In many circumstances, it may constitute part of the substrate itself.

This concept represents an important shift in perspective. The traditional model assumes that inflammation contributes to fibrosis and that fibrosis subsequently generates conduction abnormalities. Emerging evidence suggests a more direct relationship. Cytokines, immune cells, and inflammatory signaling pathways can modify electrophysiological behavior independently of structural remodeling. Consequently, electrical instability may arise not only from the architecture of diseased myocardium but also from its immunological state.

Equally important are the effects of inflammation on intercellular communication. Efficient electrical conduction depends on tightly coordinated coupling between adjacent cardiomyocytes through gap junctions, predominantly composed of connexin-43 (Cx43). Inflammatory signaling disrupts Cx43 expression, phosphorylation, and localization, promoting electrical uncoupling, conduction slowing, and increased susceptibility to ventricular arrhythmias [218,219]. Inflammatory signaling has been shown to disrupt connexin-43 organization and distribution, producing electrical uncoupling and localized conduction slowing even during the early stages of myocardial remodeling, before extensive fibrotic scar formation is established [220]. Such changes may help explain why arrhythmias can emerge during periods of active inflammation before irreversible structural remodeling becomes evident.

From a therapeutic perspective, this interpretation remains largely untested but potentially transformative. Current strategies focus predominantly on antiarrhythmic drugs, catheter ablation, implantable defibrillators, and treatment of underlying structural disease. Yet if inflammation directly contributes to the generation of arrhythmogenic substrates, immunomodulatory interventions may eventually be relevant in carefully selected patient populations. The goal would not be suppression of inflammation per se, but interruption of specific biological pathways responsible for electrical destabilization.

## 13. Current Limitations and Unresolved Questions in Immune Remodeling of Cardiomyopathies

Despite substantial advances in cardiovascular immunology, several fundamental questions remain unresolved. Much of the current framework linking immune remodeling to cardiomyopathy progression is derived from experimental models, whereas direct evidence in human disease remains comparatively limited. Consequently, distinguishing causal mechanisms from secondary biological responses represents one of the major challenges facing the field.

Similarly, the concept of immune-driven heterogeneity in cardiomyopathy requires further validation. Throughout this review, we discuss the possibility that distinct inflammatory programs may define biologically meaningful immunotypes. Although emerging data from single-cell transcriptomics, spatial biology, and translational studies support this concept, these categories should currently be regarded as conceptual rather than established clinical entities. Individual patients are likely to exhibit substantial overlap between multiple immune programs, and the dominant mechanisms may evolve during different stages of disease progression. Prospective studies integrating molecular profiling with clinical outcomes will be necessary before immunotype-based classification can be incorporated into routine practice.

Important uncertainties also surround therapeutic translation. Previous experience with anti-inflammatory strategies in cardiovascular disease illustrates the complexity of immune modulation. While selective targeting of pathways such as IL-1 signaling has shown promise, broader suppression of inflammatory responses has often yielded neutral or disappointing clinical outcomes. These observations suggest that effective intervention will require greater biological precision than has previously been possible. Future therapies may need to identify not only the appropriate molecular target but also the appropriate patient population and disease stage.

Another major challenge lies in bridging experimental discoveries with human pathology. Single-cell and spatial transcriptomic technologies have transformed our understanding of cellular interactions within diseased myocardium. Yet, most available datasets remain relatively small and are often derived from advanced-stage disease. Whether the cellular networks identified in explanted hearts accurately reflect earlier stages of remodeling remains uncertain. Longitudinal studies capable of tracking immune remodeling throughout disease evolution are still largely lacking.

Current evidence strongly supports the importance of immune remodeling in cardiomyopathies, but many mechanistic and translational questions remain open. The next phase of research must move beyond pathway identification toward rigorous determination of causality, validation of immune phenotypes in large patient cohorts, and development of clinically actionable biomarkers to guide precision immunomodulatory therapies.

## 14. From Mechanism to Medicine: Can Immune Biology Change Clinical Practice?

The history of cardiovascular medicine is marked by recurring attempts to translate biological insight into therapeutic progress. Some have transformed clinical practice; others, despite compelling experimental rationale, have failed to improve patient outcomes. The growing interest in immune mechanisms of cardiomyopathy must therefore be viewed within this broader historical context.

Contemporary management of cardiomyopathies continues to rely on a framework that has proved remarkably successful. Imaging, genetics, electrophysiology, and neurohormonal modulation remain the cornerstones of clinical decision-making. The emergence of immune biology does not invalidate these approaches. Rather, it highlights dimensions of disease that they do not adequately capture.

The central premise underlying much of the work discussed in this review is that patients with apparently similar cardiac phenotypes may differ substantially in the biological mechanisms driving disease progression. This concept is attractive because it offers a potential explanation for one of the most persistent observations in clinical cardiology: the extraordinary heterogeneity of patient outcomes. Yet acknowledging biological heterogeneity and translating it into clinical utility are fundamentally different challenges.

The notion of immunophenotyping represents one possible path forward. Advances in transcriptomics, proteomics, molecular imaging, and circulating biomarker analysis increasingly allow characterization of biological processes that were previously inaccessible in living patients. Such approaches may eventually permit the identification of dominant inflammatory programs within individual disease states; whether these programs prove stable, reproducible, and clinically meaningful remains to be determined.

To be clinically meaningful, immunophenotyping will require biomarkers that identify dominant immune remodeling programs in individual patients. Although no validated framework currently exists, emerging data suggest that distinct inflammatory pathways may be associated with characteristic molecular signatures detectable in blood, tissue, or advanced imaging studies. Although these candidate biomarkers remain largely investigational, they illustrate how integrating molecular profiling with conventional phenotypic assessment may enable the identification of biologically distinct patient subsets and facilitate the selection of mechanism-based therapeutic strategies, as shown in Table 4.

Importantly, the objective should not be to create increasingly complex classifications for their own sake. The value of any biological framework ultimately depends on its ability to improve clinical decision-making. An immunophenotype that does not influence prognosis, therapeutic selection, or disease monitoring remains an interesting observation rather than a useful clinical tool.

**Figure 4:**
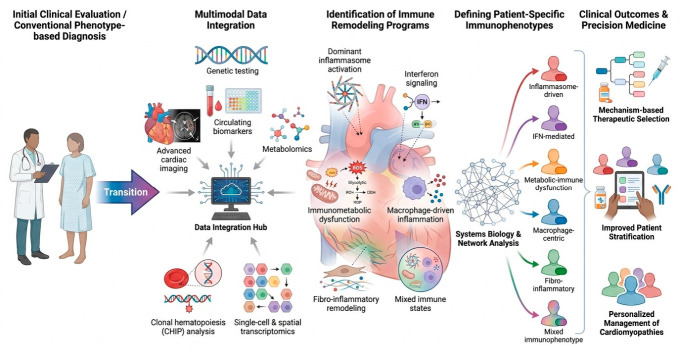
Toward Precision Immunophenotyping of Cardiomyopathies: A Translational Framework for Personalized Immune-Guided Management. The schematic emphasizes that chronic cardiac remodeling emerges from dynamic multicellular interactions rather than from isolated activation of individual signaling pathways, supporting the concept of cardiomyopathies as disorders of the cardiac immune ecosystem. Abbreviations: CCR2, C-C chemokine receptor type 2; DAMPs, damage-associated molecular patterns; ECM, extracellular matrix; IL, interleukin; MMPs, matrix metalloproteinases; NF-κB, nuclear factor kappa B; NLRP3, NOD-like receptor family pyrin domain-containing 3; ROS, reactive oxygen species; TGF-β, transforming growth factor-β; TLR, Toll-like receptor; TNF-α, tumor necrosis factor-α. Arrow notation: Solid black arrows indicate activation or progression between consecutive stages of immune remodeling following myocardial injury. Arrows connecting cardiomyocytes, immune cells, fibroblasts, endothelial cells, and extracellular matrix represent intercellular communication mediated by cytokines, chemokines, growth factors, and danger-associated molecular patterns. Bidirectional arrows indicate reciprocal interactions among immune and stromal cell populations that amplify inflammatory signaling and reinforce maladaptive remodeling. Circular or feedback arrows denote self-perpetuating inflammatory circuits in which persistent immune activation, fibroblast activation, endothelial dysfunction, and extracellular matrix remodeling continuously sustain one another. Terminal arrows indicate progression toward diffuse myocardial fibrosis, electrical remodeling, ventricular dysfunction, and heart failure. Created with FigureLabs by Urbanowicz, T (2026), ID: FL-PUB-20260628-VVKFIQ.

To facilitate translation of the concepts discussed throughout this review into a clinically relevant framework, Figure 4 summarizes a potential strategy for integrating conventional cardiovascular assessment with emerging immune profiling technologies. Rather than representing an established clinical algorithm, the proposed model illustrates how multimodal biological characterization may ultimately support individualized immunophenotyping and guide the development of mechanism-based therapeutic approaches.

Growing mechanistic insight into immune remodeling has generated numerous potential therapeutic targets across the inflammatory cascade. While most remain under investigation and their clinical utility has yet to be established, these approaches illustrate how advances in cardiovascular immunology may eventually translate into more biologically tailored treatment strategies. Table 5 summarizes selected targets and their current translational status.

Advances in molecular profiling, single-cell technologies, and spatial biology increasingly reveal dimensions of disease that are not captured by conventional clinical classifications. Rather than proposing a replacement for established diagnostic frameworks, the emerging concept of immunophenotyping may provide an additional layer of biological information to refine patient stratification and therapeutic decision-making.

The experience of other disciplines may nevertheless be instructive. Oncology has undergone a gradual transition from morphology-based classification to molecular characterization. Similar developments have occurred in rheumatology and hematology. In each case, deeper biological understanding preceded therapeutic innovation. Cardiovascular medicine may be approaching a comparable moment, although the complexity of myocardial disease and the relative inaccessibility of cardiac tissue create unique challenges.

The most realistic expectation is not the replacement of existing paradigms but their refinement. Future patient assessment may incorporate immune activity alongside genotype, imaging phenotype, fibrosis burden, and electrophysiological risk. Such integration would not represent a departure from precision medicine but rather its natural extension.

Whether this vision ultimately becomes clinical reality will depend less on the sophistication of emerging technologies than on their ability to answer practical clinical questions. The challenge for the coming decade is therefore not simply to describe immune remodeling with increasing resolution, but to determine when and how this knowledge can meaningfully alter patient care.

## 15. Critical Appraisal and Remaining Challenges

As interest in cardiovascular immunology continues to expand, it is important to maintain a balanced perspective regarding both the strengths and limitations of the current evidence. Many of the concepts discussed throughout this review are supported by substantial experimental data and increasingly sophisticated analytical approaches. At the same time, several remain at an early stage of clinical validation.

Perhaps the most important limitation concerns the relationship between association and causation. Inflammatory signatures are consistently observed in failing myocardium, but their presence alone does not establish a causal role in disease progression. The heart responds to injury through a complex series of adaptive and maladaptive mechanisms, many of which involve immune activation. Distinguishing protective responses from pathogenic ones remains one of the central challenges in the field.

This issue is particularly relevant when interpreting findings generated through modern omics technologies. Single-cell sequencing, spatial transcriptomics, and large-scale molecular profiling have dramatically improved our ability to describe biological systems. However, these approaches primarily identify associations. They reveal which cells are present, which pathways are activated, and which interactions are occurring, but they do not necessarily establish which processes are driving disease. As a consequence, increasing biological resolution does not automatically translate into mechanistic certainty.

A second limitation relates to the predominance of experimental evidence. Much of our current understanding of inflammasome activation, trained immunity, cGAS–STING signalling, and immune–fibroblast interactions originates from animal models. These systems have been invaluable for hypothesis generation, yet cardiovascular research has repeatedly demonstrated that findings derived from experimental models may not fully recapitulate human disease. Differences in lifespan, genetics, environmental exposures, and disease chronicity complicate direct translation.

The concept of immunotypes warrants particular caution. The existence of biologically distinct inflammatory states is increasingly plausible, but the boundaries separating such states remain poorly defined. It is entirely possible that immune responses exist along continuous spectra rather than within discrete categories. Furthermore, immune activity is dynamic and may evolve during disease progression, raising questions regarding the stability of any proposed classification system.

Therapeutic translation presents an additional challenge. The history of cardiovascular inflammation research contains numerous examples of promising biological targets that failed to deliver meaningful clinical benefit. Broad immunosuppression has generally produced disappointing results, while more selective approaches have yielded mixed outcomes. These experiences underscore a recurring lesson: biological plausibility alone is insufficient to guarantee therapeutic success.

Finally, there is a risk that enthusiasm for immune mechanisms may inadvertently promote a new form of reductionism. Cardiomyopathies are multifactorial disorders shaped by genetic architecture, biomechanical stress, metabolism, neurohormonal activation, environmental influences, and immune responses. None of these processes operates in isolation. The goal should therefore not be to replace cardiomyocyte-centred models with immune-centred models, but rather to develop a more integrated understanding of disease biology.

Despite these limitations, the field has reached a point at which immune mechanisms can no longer be considered peripheral to the study of cardiomyopathies. Whether they ultimately redefine disease classification or simply enrich existing frameworks remains uncertain. What is clear, however, is that future progress will require careful integration of mechanistic insight, translational research, and clinical validation. The most important advances are likely to arise not from any single pathway or technology, but from a more comprehensive understanding of how cellular, immunological, and structural processes interact throughout the course of disease.

## 16. Conclusions

The history of cardiomyopathy research can be viewed as a progressive expansion of biological perspective. Early models focused primarily on the cardiomyocyte as the principal determinant of disease. Subsequent advances highlighted the importance of neurohormonal activation, extracellular matrix remodeling, and genetic architecture. Each of these developments contributed substantially to our understanding of myocardial disease and continues to shape contemporary clinical practice. Yet none fully explains the remarkable heterogeneity that characterizes cardiomyopathies in the clinic.

A recurring theme throughout this review is that myocardial injury alone is often insufficient to explain disease progression. Equally important is the manner in which that injury is interpreted by the surrounding cellular environment. The diseased heart is not merely a collection of dysfunctional cardiomyocytes but a dynamic multicellular system in which immune cells, fibroblasts, endothelial cells, vascular structures, and hematopoietic organs continuously interact. Within this framework, inflammation is no longer viewed solely as a secondary consequence of tissue damage. Instead, it emerges as an active participant in the processes that determine whether injury resolves, stabilizes, or progresses toward fibrosis, electrical instability, and heart failure.

Recent advances in cardiovascular immunology have revealed a remarkable degree of complexity within these responses. Innate immune sensing pathways, inflammasome activation, interferon signalling, immunometabolic reprogramming, trained immunity, and cardio–bone marrow communication collectively influence the biological trajectory of disease. At the same time, single-cell and spatial technologies have demonstrated that remodeling is not a diffuse phenomenon but rather a highly organized process occurring within specific cellular microenvironments. These observations increasingly challenge the notion that cardiomyopathies can be fully understood through structural or genetic classifications alone.

Importantly, the emerging role of immune mechanisms should not be interpreted as a replacement for established paradigms. Cardiomyopathies remain multifactorial disorders shaped by genetic predisposition, biomechanical forces, metabolic disturbances, neurohormonal activation, environmental exposures, and immune responses. The contribution of immunology lies not in superseding these factors but in providing a framework through which their interactions can be better understood. The future of the field will likely depend on integrating these dimensions rather than prioritizing any one of them in isolation.

Whether immune biology will ultimately transform clinical management remains an open question. The field is still in a transitional phase in which mechanistic discovery has advanced more rapidly than therapeutic translation. Many proposed pathways remain incompletely validated in human disease, and the clinical relevance of emerging concepts such as immunophenotyping has yet to be established. Nevertheless, the direction of current evidence is difficult to ignore. The growing ability to characterize cellular states, tissue architecture, and molecular interactions within diseased myocardium is steadily revealing aspects of disease biology that were previously inaccessible.

Perhaps the most important consequence of these developments is conceptual. Cardiomyopathies are increasingly being recognized not simply as diseases of contractile dysfunction, fibrosis, or genetic abnormalities, but as disorders of a dysregulated cellular ecosystem. Disease progression reflects not only the nature of the initial insult but also the biological response that follows it. Understanding this response—and the factors that govern its transition from adaptive repair to maladaptive remodeling—may represent one of the most important challenges in cardiovascular research over the coming decade.

The ultimate value of this evolving perspective will not be measured by the number of pathways identified or technologies developed, but by its ability to improve patients’ lives. If the growing understanding of immune remodeling leads to more accurate risk stratification, more precise therapeutic selection, and a deeper understanding of disease heterogeneity, then cardiovascular immunology may ultimately contribute not merely a new layer of biological complexity, but a meaningful advance in the care of patients with cardiomyopathies and heart failure.

## Figures and Tables

**Table 1:** Cardiac Cell Types Participating in the Immune Ecosystem.

Cell Population	Physiological Role in the Healthy Heart	Contribution to Pathological Remodeling	Representative Mediators/Functions	References
Cardiomyocytes	Generate contractile force and maintain cardiac output; communicate with neighboring cells through electrical and paracrine signaling	Release DAMPs during stress or injury, initiating innate immune activation and recruitment of inflammatory cells	ATP, mitochondrial DNA, HMGB1, reactive oxygen species (ROS)	[36,37,38,39]
Resident macrophages (CCR2−)	Maintain tissue homeostasis through efferocytosis, angiogenesis support, extracellular matrix surveillance, and regulation of electrical conduction	Depletion or replacement by inflammatory populations contributes to loss of immune homeostasis and impaired repair responses	IL-10, growth factors, efferocytosis, tissue repair signaling	[40,41]
Monocyte-derived macrophages (CCR2+)	Limited presence under physiological conditions	Amplify inflammation, promote fibroblast activation, extracellular matrix remodeling, and fibrosis	IL-1β, TNF-α, IL-6, chemokines	[42]
Regulatory T cells (Tregs)	Maintain immune tolerance and suppress excessive inflammatory responses	Reduced activity may facilitate chronic inflammation, fibrosis, and ventricular dysfunction	IL-10, TGF-β, immune suppression	[43,44]
Conventional T lymphocytes	Immune surveillance and adaptive immune responses	Participate in chronic inflammation, cytokine production, and modulation of macrophage and fibroblast activity	IFN-γ, TNF-α, adaptive immune signaling	[45,46]
Cardiac fibroblasts	Maintain extracellular matrix integrity and provide structural support	Differentiate into activated fibroblasts/myofibroblasts, producing excess extracellular matrix and perpetuating inflammatory signaling	Collagens, TGF-β, IL-6, CCL2	[47,48]
Myofibroblasts	Rare in healthy myocardium	Major effector cells of fibrosis and scar formation	Collagen I/III, periostin, fibronectin	[49,50,51]
Endothelial cells	Regulate vascular integrity, perfusion, and leukocyte trafficking	Promote inflammatory cell recruitment and contribute to vascular dysfunction during remodeling	Adhesion molecules, chemokines, nitric oxide signaling	[52,53]
Dendritic cells	Antigen presentation and immune surveillance	Facilitate activation of adaptive immune responses and inflammatory amplification	Antigen presentation, cytokine production	[54,55]
Hematopoietic stem and progenitor cells	Sustain physiological hematopoiesis	Can undergo inflammatory reprogramming, contributing to trained immunity and enhanced myelopoiesis	Myeloid cell production, inflammatory memory	[56,57]
Neutrophils	First-line innate immune defense against injury and infection	Release proteases, ROS, and inflammatory mediators that can exacerbate tissue injury and remodeling	NETs, ROS, proteolytic enzymes	[58,59]

Abbreviations: CCR2, C-C chemokine receptor type 2; DAMPs, damage-associated molecular patterns; HMGB1, high-mobility group box 1; IFN-γ, interferon-γ; IL, interleukin; NETs, neutrophil extracellular traps; ROS, reactive oxygen species; TGF-β, transforming growth factor-β; TNF-α, tumor necrosis factor-α.

**Table 2:** Major Immune Mechanisms Involved in Cardiomyopathy Remodeling.

Immune Mechanism	Principal Trigger(s)	Key Molecular Components	Major Biological Effects	Representative Cardiomyopathies/Clinical Settings	References
DAMP signaling	Cardiomyocyte stress, necrosis, mitochondrial dysfunction, mechanical injury	ATP, HMGB1, mitochondrial DNA, extracellular RNA, TLRs, NF-κB	Initiation of innate immune activation, cytokine production, monocyte recruitment	DCM, myocarditis, ACM, ischemic and metabolic cardiomyopathies	[60,62,63,65,67,69,154]
NLRP3 inflammasome activation	Mitochondrial injury, ROS generation, ATP release, potassium efflux, calcium overload	NLRP3, ASC, caspase-1, IL-1β, IL-18	Pyroptosis, amplification of inflammation, fibrosis, ventricular remodeling	DCM, ACM, diabetic cardiomyopathy, heart failure	[71,73,75,155]
Immunometabolic reprogramming	Metabolic stress, obesity, diabetes, mitochondrial dysfunction, hypoxia	HIF-1α, mTOR, AMPK, succinate, glycolytic pathways	Sustained inflammatory macrophage activation, endothelial dysfunction, profibrotic signaling	HFpEF, metabolic cardiomyopathy, obesity-associated heart disease	[91,98,102]
Trained immunity	Recurrent inflammatory stimulation, chronic tissue injury, metabolic stress	Epigenetic remodeling, H3K4me3, H3K27ac, mevalonate pathway, fumarate, succinate	Persistent inflammatory responsiveness independent of the initiating insult	Chronic heart failure, post-myocarditis remodeling, inherited cardiomyopathies	[103,156]
cGAS–STING signaling	Cytoplasmic accumulation of nuclear or mitochondrial DNA	cGAS, cGAMP, STING, TBK1, IRF3, type I interferons	Interferon signaling, macrophage recruitment, fibroblast activation, fibrosis	LMNA cardiomyopathy, myocarditis, pressure-overload and age-related heart failure	[107,109,157,158]
Cardio–bone marrow axis	Chronic myocardial injury and inflammatory signaling	Cytokines, chemokines, hematopoietic stem and progenitor cells	Enhanced myelopoiesis, increased inflammatory monocyte production, sustained systemic inflammation	Chronic heart failure, advanced cardiomyopathies	[159,160]
Clonal hematopoiesis (CHIP)	Somatic mutations in hematopoietic stem cells during aging	TET2, DNMT3A, ASXL1, JAK2	Exaggerated innate immune activation, enhanced inflammasome signaling, accelerated fibrosis	Heart failure, age-related cardiovascular disease, adverse remodeling	[161,162]
Fibroblast–immune cell crosstalk	Chronic inflammatory activation and tissue stress	TGF-β, IL-6, CCL2, macrophage-derived cytokines	Myofibroblast activation, extracellular matrix deposition, persistent remodeling	Present across virtually all cardiomyopathy subtypes	[163]

Abbreviations: ACM, arrhythmogenic cardiomyopathy; ASC, apoptosis-associated speck-like protein containing a CARD; CHIP, clonal hematopoiesis of indeterminate potential; DAMPs, damage-associated molecular patterns; DCM, dilated cardiomyopathy; HMGB1, high-mobility group box 1; HFpEF, heart failure with preserved ejection fraction; HIF-1α, hypoxia-inducible factor-1α; IRF3, interferon regulatory factor 3; NF-κB, nuclear factor kappa B; ROS, reactive oxygen species; STING, stimulator of interferon genes; TBK1, TANK-binding kinase 1; TLR, Toll-like receptor.

**Table 3:** Immune Remodeling Across Major Cardiomyopathy Phenotypes *.

Cardiomyopathy Phenotype	Primary Disease Driver	Dominant immune Mechanism(s)	Characteristic Remodeling Features	Potential Immunotype	References
Dilated cardiomyopathy (DCM)	Genetic variants (e.g., TTN, FLNC), viral injury, toxic exposure, autoimmune or idiopathic factors	DAMP signaling, macrophage activation, NLRP3 inflammasome activation, fibroblast–immune crosstalk	Ventricular dilatation, systolic dysfunction, diffuse fibrosis, chronic inflammation	Macrophage/inflammasome-dominant	[188,189]
Arrhythmogenic cardiomyopathy (ACM)	Desmosomal gene defects (e.g., DSP, PKP2, DSG2, DSC2)	Innate immune activation, inflammasome signaling, recurrent inflammatory episodes	Fibrofatty replacement, ventricular arrhythmias, inflammatory “hot phases”	Inflammasome-dominant	[190,191]
LMNA-associated cardiomyopathy	Nuclear envelope dysfunction caused by LMNA variants	cGAS–STING activation, type I interferon signaling, macrophage recruitment	Early conduction disease, ventricular arrhythmias, progressive fibrosis and heart failure	Interferon-dominant	[192,193]
Inflammatory cardiomyopathy/Myocarditis	Viral infection, autoimmunity, immune-mediated injury	Innate and adaptive immune activation, cytokine signaling, inflammasome activation	Myocyte injury, inflammatory infiltrates, variable fibrosis, ventricular dysfunction	Inflammatory mixed phenotype	[194,195]
Metabolic cardiomyopathy	Obesity, insulin resistance, diabetes mellitus, metabolic syndrome	Immunometabolic reprogramming, chronic low-grade inflammation, macrophage activation	Myocardial stiffness, fibrosis, microvascular dysfunction, metabolic remodeling	Immunometabolic-dominant	[196,197]
Heart failure with preserved ejection fraction (HFpEF)	Aging, obesity, hypertension, diabetes, chronic kidney disease	Systemic inflammation, endothelial dysfunction, immunometabolic activation, monocyte recruitment	Diastolic dysfunction, microvascular rarefaction, interstitial fibrosis	Immunometabolic/fibrotic	[198,199]
Ischemic cardiomyopathy	Myocardial infarction and chronic ischemic injury	DAMP signaling, inflammasome activation, monocyte–macrophage recruitment	Scar formation, adverse ventricular remodeling, progressive heart failure	Post-injury inflammatory	[200,201]
Anthracycline-induced cardiomyopathy	Chemotherapy-related cardiotoxicity	Oxidative stress, mitochondrial injury, DAMP release, inflammasome activation	Progressive ventricular dysfunction, fibrosis, impaired myocardial recovery	Stress-induced inflammatory	[202,203]
Advanced heart failure (multiple etiologies)	Chronic myocardial injury and remodeling	Trained immunity, cardio–bone marrow axis activation, CHIP-associated inflammation	Persistent systemic inflammation, progressive fibrosis, worsening ventricular function	Systemic inflammatory/remodeling phenotype	[204]

Abbreviations: ACM, arrhythmogenic cardiomyopathy; CHIP, clonal hematopoiesis of indeterminate potential; DAMPs, damage-associated molecular patterns; DCM, dilated cardiomyopathy; HFpEF, heart failure with preserved ejection fraction; LMNA, lamin A/C. * The proposed immunotypes represent conceptual categories intended to illustrate dominant immune programs identified in experimental and translational studies. They are not mutually exclusive and should not be interpreted as established clinical classifications. Individual patients may exhibit overlap between multiple immune remodeling pathways.

**Table 4:** Candidate Biomarkers for Emerging Immune Remodeling Programs in Cardiomyopathy.

Dominant Immune Program	Candidate Biomarkers	Biological Interpretation	Current Level of Evidence	References
Inflammasome-dominant remodeling	hsCRP, IL-1β, IL-18, inflammasome-related gene signatures	Reflects activation of NLRP3 signaling, pyroptosis, and cytokine-mediated inflammation	Clinical proof-of-concept; translational studies ongoing	[221,222]
Interferon-dominant remodeling	Type I interferon gene signatures, CXCL10, interferon-stimulated genes (ISGs)	Indicates activation of cGAS–STING signaling and chronic interferon responses	Predominantly preclinical and translational evidence	[223,224]
Immunometabolic remodeling	Insulin resistance indices, adipokines (leptin, adiponectin), metabolomic profiles, inflammatory cytokines	Reflects metabolic stress–driven immune activation and macrophage reprogramming	Clinical and translational evidence	[225,226]
Macrophage-driven inflammatory remodeling	CCL2, circulating monocyte activation markers, inflammatory transcriptomic signatures	Suggests enhanced monocyte recruitment and macrophage-mediated tissue remodeling	Experimental and early translational evidence	[227,228]
CHIP-associated remodeling	Somatic mutations in *TET2*, *DNMT3A*, *ASXL1*, or *JAK2*; inflammatory biomarkers	Indicates hematopoietic-driven amplification of inflammatory responses	Strong epidemiological and experimental evidence	[229,230]
Fibro-inflammatory remodeling	Galectin-3, soluble ST2, periostin, collagen turnover biomarkers (PINP, PIIINP, CITP)	Reflects active fibroblast activation, extracellular matrix remodeling, and fibrosis progression	Experimental and early clinical evidence	[231]
Adaptive immune–associated remodeling	Autoantibodies, T-cell activation markers, cytokine profiles (IFN-γ, TNF-α)	Suggests participation of adaptive immune pathways and chronic immune activation	Emerging translational evidence	[232,233]
Systemic inflammatory remodeling	hsCRP, IL-6, TNF-α, neutrophil-to-lymphocyte ratio, circulating inflammatory proteomic signatures	Reflects persistent low-grade inflammation associated with advanced heart failure and multisystem remodeling	Clinical observational evidence	[234,235]

Abbreviations: ASXL1, additional sex combs-like 1; CCL2, C-C motif chemokine ligand 2; CHIP, clonal hematopoiesis of indeterminate potential; CITP, carboxy-terminal telopeptide of type I collagen; cGAS, cyclic GMP–AMP synthase; CXCL10, C-X-C motif chemokine ligand 10; DNMT3A, DNA methyltransferase 3 alpha; hsCRP, high-sensitivity C-reactive protein; IFN-γ, interferon-γ; IL, interleukin; ISGs, interferon-stimulated genes; JAK2, Janus kinase 2; NLRP3, NOD-, LRR-, and pyrin domain-containing protein 3; PINP, procollagen type I N-terminal propeptide; PIIINP, procollagen type III N-terminal propeptide; ST2, suppression of tumorigenicity 2; STING, stimulator of interferon genes; TET2, ten-eleven translocation 2; TNF-α, tumor necrosis factor-α.

**Table 5:** Emerging immunomodulatory therapeutic targets *.

Therapeutic Target/Pathway	Representative Agent(s)	Mechanism of Action	Current Evidence Status	Potential Clinical Application	References
IL-1 signaling	Anakinra, Canakinumab	Inhibition of IL-1-mediated inflammatory responses downstream of inflammasome activation	Clinical studies and randomized trials; proof-of-concept established in cardiovascular disease	Inflammatory cardiomyopathy, myocarditis, selected heart failure phenotypes	[236,237]
NLRP3 inflammasome	MCC950, dapansutrile (OLT1177)	Direct inhibition of inflammasome assembly and IL-1β/IL-18 production	Preclinical and early clinical development	Inflammasome-driven remodeling, fibrosis, ventricular dysfunction	[238,239]
cGAS–STING pathway	Experimental STING inhibitors, cGAS inhibitors	Suppression of cytosolic DNA sensing and interferon signaling	Primarily preclinical	LMNA cardiomyopathy, interferon-driven remodeling, inflammatory heart failure	[240,241]
TNF-α signaling	Infliximab, etanercept	Neutralization of TNF-α-mediated inflammatory signaling	Clinical trials largely unsuccessful in heart failure	Limited current role; important historical target	[242,243]
IL-6 signaling	Tocilizumab	Blockade of IL-6 receptor-mediated inflammatory pathways	Early clinical and translational evidence	Selected inflammatory and immune-mediated cardiomyopathies	[244,245]
Immunometabolic pathways	SGLT2 inhibitors, GLP-1 receptor agonists	Modulation of cellular metabolism, oxidative stress, and inflammatory activation	Established clinical benefit in heart failure and metabolic disease	HFpEF, metabolic cardiomyopathy, obesity-related cardiac disease	[246,247]
Fibroblast activation/TGF-β signaling	Pirfenidone, anti-TGF-β strategies, integrin inhibitors	Reduction in fibroblast activation and extracellular matrix deposition	Preclinical and early clinical studies	Progressive myocardial fibrosis and adverse remodeling	[248,249]
Chemokine-mediated monocyte recruitment	CCR2 antagonists, CCL2-targeted therapies	Limitation of inflammatory monocyte infiltration into the myocardium	Experimental and early translational studies	Macrophage-driven remodeling and chronic inflammation	[250,251]
Epigenetic regulation of trained immunity	Bromodomain inhibitors, histone-modifying enzyme inhibitors	Reversal of inflammatory memory and maladaptive immune reprogramming	Experimental	Chronic heart failure and persistent inflammatory states	[251,252]
Clonal hematopoiesis-associated inflammation (CHIP)	Precision anti-inflammatory approaches (investigational)	Targeting mutation-associated inflammatory pathways	Emerging translational field	CHIP-positive patients with accelerated cardiovascular disease	[176,252]
Regulatory immune cell enhancement	Treg-based therapies, low-dose IL-2 (investigational)	Restoration of immune tolerance and suppression of excessive inflammation	Early-stage experimental development	Autoimmune and inflammatory cardiomyopathies	[177,253]

Abbreviations: HFpEF, heart failure with preserved ejection fraction; LMNA, lamin A/C. * The proposed immunotypes represent conceptual categories intended to illustrate dominant immune programs identified in experimental and translational studies. They are not mutually exclusive and should not be interpreted as established clinical classifications. Individual patients may exhibit overlap between multiple immune remodeling pathways.

## Data Availability

No new data were created for this manuscript.

## Abbreviations

The following abbreviations are used in this manuscript:

ACM	Arrhythmogenic cardiomyopathy
AMPK	AMP-activated protein kinase
ASC	Apoptosis-associated speck-like protein containing a CARD
ATP	Adenosine triphosphate
CCL2	C-C motif chemokine ligand 2
CCR2	C-C chemokine receptor type 2
cGAMP	Cyclic guanosine monophosphate–adenosine monophosphate
cGAS	Cyclic GMP–AMP synthase
CHIP	Clonal hematopoiesis of indeterminate potential
DAMPs	Damage-associated molecular patterns
DCM	Dilated cardiomyopathy
DNA	Deoxyribonucleic acid
DSP	Desmoplakin
DSC2	Desmocollin-2
DSG2	Desmoglein-2
ECM	Extracellular matrix
FLNC	Filamin C
GLP-1RA	Glucagon-like peptide-1 receptor agonist
HF	Heart failure
HFpEF	Heart failure with preserved ejection fraction
HIF-1α	Hypoxia-inducible factor-1 alpha
HMGB1	High-mobility group box 1
HSPCs	Hematopoietic stem and progenitor cells
IFN-γ	Interferon gamma
IL	Interleukin
IL-1β	Interleukin-1 beta
IL-6	Interleukin-6
IL-10	Interleukin-10
IL-18	Interleukin-18
IRF3	Interferon regulatory factor 3
JAK2	Janus kinase 2
LMNA	Lamin A/C gene
mTOR	Mechanistic target of rapamycin
NETs	Neutrophil extracellular traps
NF-κB	Nuclear factor kappa B
NLRP3	NOD-like receptor family pyrin domain-containing 3
PKP2	Plakophilin-2
PRRs	Pattern-recognition receptors
RNA	Ribonucleic acid
ROS	Reactive oxygen species
SGLT2i	Sodium–glucose cotransporter-2 inhibitors
STING	Stimulator of interferon genes
TBK1	TANK-binding kinase 1
TET2	Tet methylcytosine dioxygenase 2
TGF-β	Transforming growth factor beta
TLR	Toll-like receptor
TNF-α	Tumor necrosis factor alpha
Tregs	Regulatory T cells
TTN	Titin
